# *GABRA1* frameshift variants impair GABAA receptor proteostasis

**DOI:** 10.1101/2024.11.28.625971

**Published:** 2024-11-29

**Authors:** Marnie P. Williams, Ya-Juan Wang, Jing-Qiong Kang, Ting-Wei Mu

## Abstract

The gamma-aminobutyric acid type A receptor (GABA _A_ R) is the most common inhibitory neurotransmitter-gated ion channel in the central nervous system. Pathogenic variants in genes encoding GABA _A_ R subunits can cause receptor dysfunction and lead to genetic epilepsy. Frameshift variants in these genes can result in a premature termination codon, producing truncated receptor subunit variants. However, the molecular mechanism as well as functional implications of these frameshift variants remains inadequately characterized. This study focused on four clinical frameshift variants of the α _1_ subunit of GABA _A_ R (encoded by the *GABRA1* gene): K401fs (c.1200del), S326fs (c.975del), V290fs (c.869_888del), and F272fs (c.813del). These variants result in the loss of one to three transmembrane helices, whereas wild type α1 has four transmembrane helices. Therefore, these variants serve as valuable models to evaluate membrane protein biogenesis and proteostasis deficiencies. In HEK293T cells, all four frameshift variants exhibit significantly reduced trafficking to the cell surface, resulting in essentially non-functional ion channels. However, the severity of proteostasis deficiency varied among these four frameshift variants, presumably due to their specific transmembrane domain deletions. The variant α1 subunits exhibited endoplasmic reticulum (ER) retention and activated the unfolded protein response (UPR) to varying extents. Our findings revealed that these frameshift variants of *GABRA1* utilize overlapping yet distinct molecular mechanisms to impair proteostasis, providing insights into the pathogenesis of GABA _A_ R-associated epilepsy.

## Full Text Availability

The license terms selected by the author(s) for this preprint version do not permit archiving in PMC. The full text is available from the preprint server.

