# Supplementary material for: GABRA1 frameshift variants impair GABAA receptor proteostasis
Source: bioRxiv. 2025 Jan 8:2024.11.28.625971. Originally published 2024 Nov 29. Preprint. [Version 2] doi: 10.1101/2024.11.28.625971 (PMC11623673; doi:10.1101/2024.11.28.625971)
Supplement: 1 [file NIHPP2024.11.28.625971V2-supplement-1.pdf]

bioRxiv preprint doi: https://doi.org/10.1101/2024.11.28.625971; this version posted January 8, 2025. The copyright holder for this preprint (which was not certified by peer review) is the author/funder, who has granted bioRxiv a license to display the preprint in perpetuity. It is made available under aCC-BY 4.0 International license.

Supplemental Tables

Table S1. List of qPCR primers that were utilized in this study.

Gene	Forward Primer 5'– 3'	Reverse Primer 5'– 3'
RPLP2	TTGGACAGCGTGGGTATCG	CCAGCAGGTACACTGGCAA
GABRA1	GTCACCAGTTTCGGACCCG	AACCGGAGGACTGTCATAGGT
BiP (HspA5)	CTGTCCAGGCTGGTGTGCTCT	CTTGGTAGGCACCACTGTGTTC
ERdj3 (DNAJB11)	ACGCTGGAAGTAGAAATAGAGCC	TCGGAACCGTAAATCTCCAGGC
CHOP	GAACGGCTCAAGCAGGAAATC	TTCACCATTCGGTCAATCAGAG

Table S2. List of statistical tests utilized in this study.

Figure Panel	Assay	Statistical test	Adjusted P-value
Figure 1D	Total α_1 expression	One-way ANOVA test, Dunnett's post-hoc test	WT vs $K401_{fs}$; p = 0.5060<br>WT vs $S326_{fs}$; p = 0.0057<br>WT vs $V290_{fs}$; p = <0.0001<br>WT vs $F272_{fs}$; p = 0.1172
Figure 1E	Oligomer to monomer α_1 ratio	One-way ANOVA test, Dunnett's post-hoc test	WT vs $K401_{fs}$; p = 0.1521<br>WT vs $S326_{fs}$; p = 0.0045<br>WT vs $V290_{fs}$; p = 0.0029<br>WT vs $F272_{fs}$; p = 0.0018
Figure 2A	Surface biotinylation	One-way ANOVA test, Dunnett's post-hoc test	WT vs $K401_{fs}$; p = 0.0009<br>WT vs $S326_{fs}$; p = 0.0018<br>WT vs $V290_{fs}$; p = 0.0014<br>WT vs $F272_{fs}$; p = 0.0004
Figure 2B	Immunocytochemistry –surface vs intracellular α_1	One-way ANOVA test, Dunnett's post-hoc test	WT vs $K401_{fs}$; p = <0.0001<br>WT vs $S326_{fs}$; p = <0.0001<br>WT vs $V290_{fs}$; p = <0.0001<br>WT vs $F272_{fs}$; p = <0.0001
Figure 2C	Automated patch clamp recordings	One-way ANOVA test, Dunnett's post-hoc test	WT vs $K401_{fs}$; p = <0.0001<br>WT vs $S326_{fs}$; p = <0.0001<br>WT vs $V290_{fs}$; p = <0.0001<br>WT vs $F272_{fs}$; p = <0.0001
Figure 3A	Endo H digestion	One-way ANOVA test, Dunnett's post hoc test	WT vs $K401_{fs}$; p = <0.0001<br>WT vs $S326_{fs}$; p = <0.0001<br>WT vs $V290_{fs}$; p = <0.0001<br>WT vs $F272_{fs}$; p = <0.0001
Figure 3B	Immunocytochemistry –α_1 and calnexin co-	One-way ANOVA test,	WT vs $K401_{fs}$; p = <0.0001<br>WT vs $S326_{fs}$; p = <0.0001

bioRxiv preprint doi: https://doi.org/10.1101/2024.11.28.625971; this version posted January 8, 2025. The copyright holder for this preprint (which was not certified by peer review) is the author/funder, who has granted bioRxiv a license to display the preprint in perpetuity. It is made available under aCC-BY 4.0 International license.

	localization	Dunnett's post hoc test	WT vs $V290_{fs}$; p = 0.0041<br>WT vs $F272_{fs}$; p = 0.0405
Figure 4A	Proteasomal vs lysosomal degradation	One-way ANOVA test, Dunnett's post-hoc test	WT: DMSO vs BafA1; p = 0.0038<br>$S326_{fs}$: DMSO vs BafA1; p = 0.0158<br>$S326_{fs}$: DMSO vs MG132; p = 0.0184<br>$F272_{fs}$: DMSO vs BafA1; p = 0.0020
Figure 4B	qPCR analysis for NMD	Unpaired t-test	$S326_{fs}$; p = 0.0166<br>$V290_{fs}$; p = <0.0001<br>$F272_{fs}$; p = 0.0090
Figure 5A	qPCR analysis for UPR activation	One-way ANOVA test, Dunnett's post-hoc test	ERdj3:<br>WT vs $K401_{fs}$; p = 0.3256<br>WT vs $S326_{fs}$; p = 0.2819<br>WT vs $V290_{fs}$; p = 0.0009<br>WT vs $F272_{fs}$; p = 0.0211<br>BiP:<br>WT vs $K401_{fs}$; p = 0.0693<br>WT vs $S326_{fs}$; p = 0.1084<br>WT vs $V290_{fs}$; p = <0.0001<br>WT vs $F272_{fs}$; p = 0.0294<br>CHOP:<br>WT vs $K401_{fs}$; p = 0.0207<br>WT vs $S326_{fs}$; p = 0.1448<br>WT vs $V290_{fs}$; p = 0.0011<br>WT vs $F272_{fs}$; p = 0.2414
Figure 5B	IRE1 inhibition	Unpaired t-test	WT: DMSO vs KIRA6; p = 0.097955<br>$K401_{fs}$: DMSO vs KIRA6; p = 0.00026<br>$S326_{fs}$: DMSO vs KIRA6; p = 0.000176<br>$V290_{fs}$: DMSO vs KIRA6; p = 0.045941<br>$F272_{fs}$: DMSO vs KIRA6; p = 0.000462
Figure 5C	PERK inhibition	Unpaired t-test	WT: DMSO vs GSK; p = 0.080790<br>$K401_{fs}$: DMSO vs GSK; p = 0.001282<br>$S326_{fs}$: DMSO vs GSK; p = 0.023712<br>$V290_{fs}$: DMSO vs GSK; p = 0.168432<br>$F272_{fs}$: DMSO vs GSK; p = 0.264301

bioRxiv preprint doi: https://doi.org/10.1101/2024.11.28.625971; this version posted January 8, 2025. The copyright holder for this preprint (which was not certified by peer review) is the author/funder, who has granted bioRxiv a license to display the preprint in perpetuity. It is made available under aCC-BY 4.0 International license.

Supplemental figure legends

Supplemental Figure S1. Inhibition of the proteasome or the lysosome. To inhibit proteasomal and lysosomal degradation, MG132 (200 nM, 24 hrs) and BafA1 (20 nM, 24 hrs) were applied, respectively. Total α_1 proteins were extracted 48 hours after transfection, followed by SDS-PAGE and Western blot to detect the ubiquitin protein (**A**) and the LC3b isoforms (**B**). β-actin was used as a loading control. Data is presented as mean ± SEM. (**C**) Cartoon of minigene constructs of human *GABRA1* containing intron 8, showing the stop codons resulting from the K401, S326, V290, and F272 deletions (indicated by the star). One-way ANOVA followed by Dunnett's test was used for statistical analysis. *, $p < 0.05$; **, $p < 0.01$; ***, $p < 0.001$, ****, $p < 0.0001$.

Supplemental Figure S2. Effect of α_1 variants on the UPR. (**A**) Thapsigargin (Tg, 1 µM, 6 hrs) was applied to HEK293T cells as a positive control of UPR activation. Relative mRNA expression of UPR targets, including BiP, ERdj3, and CHOP for negative (plain) and positive control (plain + Tg) samples (n=5-6). (**B**) KIRA6 (1 µM, 24 hrs), an IRE1 inhibitor, was applied to HEK293T cells expressing WT or frameshift α1 mutants. Total proteins were extracted 48 hours after transfection, followed by SDS-PAGE and Western blot to detect XBP1-s and confirm IRE1 inhibition (n=5). (**C**) GSK2606414 (0.5 µM, 24 hrs), a PERK inhibitor, was applied to HEK293T cells expressing WT or frameshift α1 mutants. Total proteins were extracted 48 hours post transfection, followed by SDS-PAGE and Western blot to detect the PERK and phosphorylated PERK (n=5). Proteins bands were quantified using ImageJ. β-actin was used as a loading control. Data is presented as mean ± SEM. Unpaired T-test was used for two group comparisons. *, $p < 0.05$; ***, $p < 0.001$; ****, $p < 0.0001$.

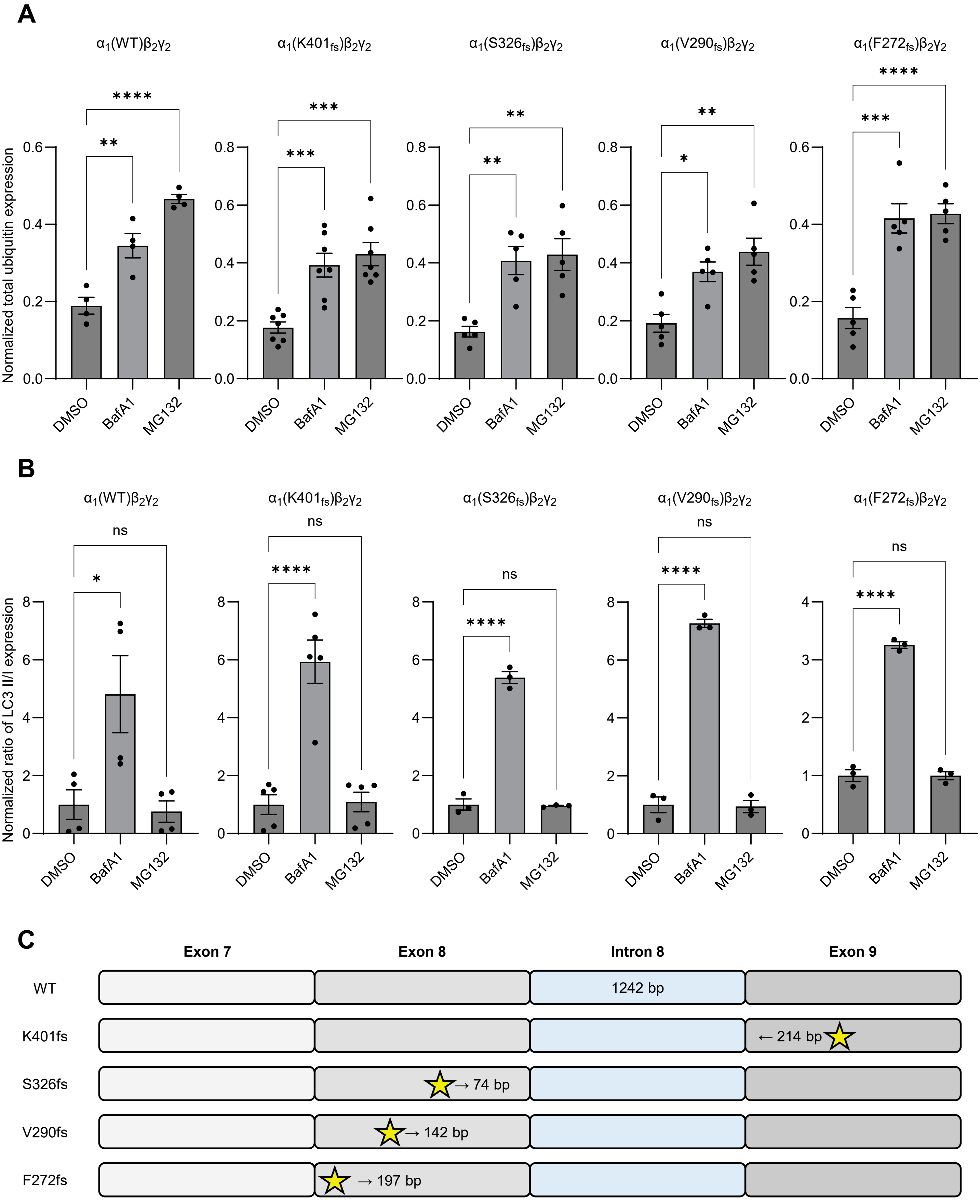

Supplemental Fig. S1

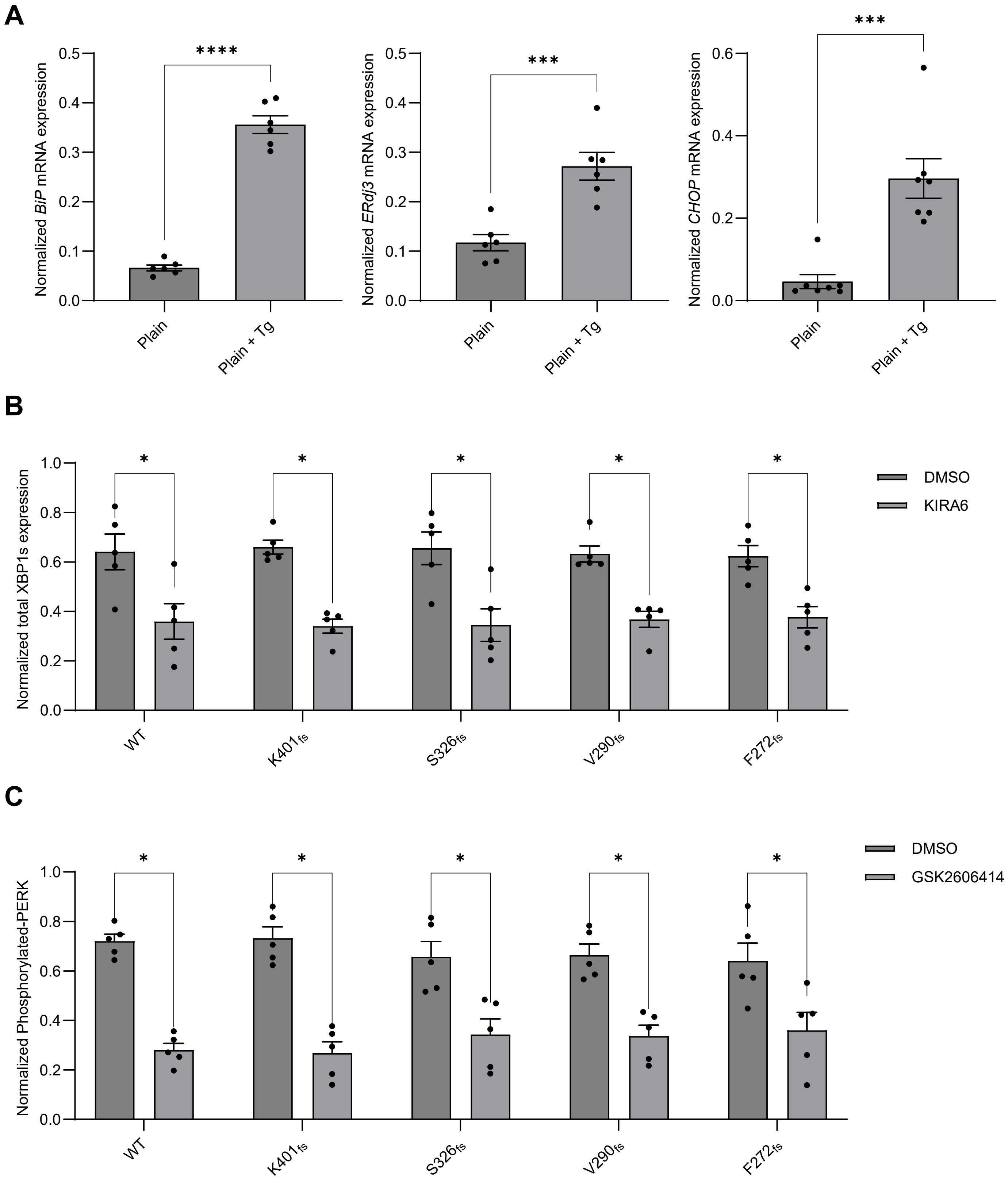

Supplemental Fig. S2
